# Phenotyping the autonomic nervous system in pregnancy using remote sensors: potential for complication prediction

**DOI:** 10.3389/fphys.2023.1293946

**Published:** 2023-11-21

**Authors:** Zahra Sharifi-Heris, Zhongqi Yang, Amir M. Rahmani, Michelle A. Fortier, Hamid Sharifiheris, Miriam Bender

**Affiliations:** ^1^ Sue and Bill Gross School of Nursing, University of California, Irvine, Irvine, CA, United States; ^2^ Department of Computer Science, University of California, Irvine, Irvine, CA, United States; ^3^ Center on Stress and Health, University of California, Irvine, Irvine, CA, United States; ^4^ Department of Computer Science, Azad University of Ahar, Ahar, Iran

**Keywords:** autonomic nervous system, healthy pregnancy, pregnancy complications, smart wearable technology, physiology

## Abstract

**Objectives:** The autonomic nervous system (ANS) plays a central role in dynamic adaptation during pregnancy in accordance with the pregnancy demands which otherwise can lead to various pregnancy complications. Despite the importance of understanding the ANS function during pregnancy, the literature lacks sufficiency in the ANS assessment. In this study, we aimed to identify the heart rate variability (HRV) function during the second and third trimesters of pregnancy and 1 week after childbirth and its relevant predictors in healthy pregnant Latina individuals in Orange County, CA.

**Materials and methods:** *N* = 16 participants were enrolled into the study from which *N* = 14 (*N* = 13 healthy and n = 1 complicated) participants proceeded to the analysis phase. For the analysis, we conducted supervised machine learning modeling including the hierarchical linear model to understand the association between time and HRV and random forest regression to investigate the factors that may affect HRV during pregnancy. A *t*-test was used for exploratory analysis to compare the complicated case with healthy pregnancies.

**Results:** The results of hierarchical linear model analysis showed a significant positive relationship between time (day) and average HRV (estimated effect = 0.06; *p* < 0.0001), regardless of being healthy or complicated, indicating that HRV increases during pregnancy significantly. Random forest regression results identified some lifestyle and sociodemographic factors such as activity, sleep, diet, and mental stress as important predictors for HRV changes in addition to time. The findings of the *t*-test indicated that the average weekly HRV of healthy and non-healthy subjects differed significantly (*p* < 0.05) during the 17 weeks of the study.

**Conclusion:** It is imperative to focus our attention on potential autonomic changes, particularly the possibility of increased parasympathetic activity as pregnancy advances. This observation may challenge the existing literature that often suggests a decline in parasympathetic activity toward the end of pregnancy. Moreover, our findings indicated the complexity of HRV prediction, involving various factors beyond the mere passage of time. To gain a more comprehensive understanding of this dynamic state, future investigations should delve into the intricate relationship between autonomic activity, considering diverse parasympathetic and sympathetic metrics, and the progression of pregnancy.

## Introduction

Various physiological changes occur during pregnancy contributing to the optimal growth and development of the fetus and help protect the mother from the pregnancy and delivery complications (Soma-Pillay et al., 2016). These changes are regulated through a non-linear complex relationship between various vital systems in the body and, to a great extent, by the autonomic nervous systems (ANS). The ANS role can be mainly explained by the adaptation of sympathetic (SNS) and parasympathetic (PNS) components and their responses to the pregnancy demands as stimuli (Waxenbaum, et al., 2019). SNS directs the body’s rapid involuntary response to various demanding situations in or outside of the body (Bruno et al., 2012) and mediates the vigilance, arousal, and activation of the bodily responses to adapt to increased metabolic needs in response to internal and external stimuli including pregnancy (Braeken., 2014). PNS, on the other hand, is responsible for the stimulation of “rest-and-digest” or “feed-and-breed” activities that occur when the body is at rest (Johnson, 2019). Sympathetic and parasympathetic systems function as complementary components to maintain the hemostasis and hemodynamic adaptation in the body.

A failure in the ANS including SNS and PNS function has been described in various diseases, both those that directly afflict the nervous system and those afflicting other organs, where they indirectly trigger or enhance pathological symptoms in the body (Zygmunt and Stanczyk, 2010). Broadly speaking, every disorder or ailment is inherently linked to disturbances or dysfunctions in autonomic regulation or innervation (Ziemssen and Siepmann, 2019). Likewise, in pregnant individuals, ANS dysfunction has been considered to be one of the main contributors to the development of some maternal and/or neonatal disorders such as hypertensive disorders and preeclampsia (Yousif et al., 2019), gestational diabetes mellitus, and unexplained recurrent pregnancy loss (Kataoka et al., 2015). This may explain the ANS role in physiological regulation helping the individuals’ body adjust to the pregnancy-related demands in order to prevent these disorders. On the other hand, for the aforementioned adverse pregnancy outcomes, evidence has identified several sociodemographic and medical history factors as potential risk factors, which are also associated with ANS dysfunction. These include age (Jandackova et al., 2016; Geovanini et al., 2020), prenatal alcohol consumption (Karpyak et al., 2014; Jurczyk et al., 2019), smoking (Harte et al., 2013; Bodin et al., 2017; Murgia et al., 2019), drug use (Nayak et al., 2020; Lu et al., 2022; Qiu et al., 2023), and body mass index (BMI) (Rodrigues and Quarto, 2018; Triggiani et al., 2019). Due to the associations between the aforementioned risk factors and pregnancy complications and ANS dysfunction, it seems that the ANS may explain the pathway between the risk factors and the pregnancy outcomes, which already indicated in ANS dysfunction (or maybe correctional but not optimal adaptation) in response to the risk factors resulting in pregnancy complications. However, the reliability of these results is questionable due to serious methodological limitations in the assessment of the ANS. Many studies have relied on short-duration (5–10 min) and non-continuous assessments (Sharifiheris et al., 2022), which is problematic considering that pregnancy is a dynamic and ever-changing condition that cannot be adequately captured through brief and episodic measurements. In order to comprehensively understand pathological and abnormal changes, it is essential to first establish a baseline of normal ANS function in healthy pregnancies. To initiate this inquiry, it is crucial to initially investigate whether a consistent pattern is present among individuals experiencing healthy pregnancies. This preliminary yet vital step will help us ascertain the feasibility of conducting a trial that compares healthy and unhealthy pregnancies, providing an opportunity to identify potential differences.

The most common tests for ANS assessment are those that evaluate the cardiovascular reflexes in response to provocative maneuvers. These tests include the orthostatic stress test, cold pressor test, deep breathing test, Valsalva maneuver, isometric hand grip test, head-up tilt test, and mental stress test (Zygmunt and Stanczyk, 2010; Ziemssen and Siepmann, 2019). Although the aforementioned ANS assessment maneuvers are widely applied in clinical settings for diagnostic purposes, the ability of these maneuvers to reflect ANS function in real life is questionable. This is due to the fact that the tests are often performed in a controlled situation in which individuals have to follow specific considerations in order to standardize the test situation for all people. For example, several hours before a test, caffeine, nicotine, alcohol, and certain medications are needed to be withheld. Additionally, tests are required to be performed in a specific environmental situation (e.g., temperature and humidity), and right before and during the test, people have to practice specific positions (standing up and lying down). These framed rules before and during the test and artificial setting for the test procedure cannot reflect one’s ANS activity in the everyday life. Specifically, since the ANS follows a 24-h cycle of circadian rhythm, it cannot be represented in any duration less than 24 h. In addition, as discussed earlier, pregnancy is an ever-changing situation that episodic assessments are unable to reflect this dynamic situation. Thus, relying on the test results seems to be tricky to make a right decision regarding the ANS function.

In the current study, heart rate variability (HRV) has been applied to assess the ANS function. HRV is one of the well-known, non-invasive ANS biomarkers that is commonly applied in the recent literature to detect various physical and psychological disorders resulted from ANS dysfunction (Duong et al., 2020). Variability of the heart rate indicates the flexibility to cope with the uncertain and changing environment through the cardiovascular system. HRV is a surrogate parameter of the ANS reflecting the complex interaction between organ systems and, specifically, the brain and the cardiovascular system to maintain hemostasis (Ernst, 2017). HRV applies the photoplethysmogram (PPG) technique and is equipped with a light source (red and infrared light-emitting diodes) and a detector to function. HRV can cover the existing literature gaps in ANS assessment. This is because PPG is embeddable in smart devices enabling continuous and long-term ANS assessment (Peng et al., 2015).

The overall purpose of this study is to determine the ANS function during normal (low risk) pregnancy across healthy pregnant populations when assessed by HRV using a tech-based wearable smart device. This investigation serves as a foundational step toward creating a screening tool that has potential to identify any abnormalities in the normal ANS patterns during pregnancy, enabling timely decision making. In this study, we will focus on the Latina population who are at more risk of developing pregnancy complications (Howell et al., 2017; Petersen et al., 2019).

## Materials and methods

### Study design

A prospective longitudinal observational study design was utilized in the current study. The study started at 22–24 gestational age (GA) and continued until 1 week after childbirth (16–19 weeks). A prospective study that is supposed to be carried out from the present time into the future is appropriate for this study because 1) our target population is required to meet specific criteria to be eligible for recruitment, 2) the dependent variable should be assessed in a specific time frame and consistently to be able to assess its pattern of changes and relationship with the independent variable, and 3) there is no existing data source that matches with the aim and requirements of the current study to carry out it in a retrospective design.

### Sample and setting

Our target population comprises pregnant individuals who currently live in southern California. Following IRB approval, we recruited the study subjects from the Manchester Clinic using purposeful sampling. Specified criteria were considered to address the research questions of the study. The inclusion criteria were as follows: 1) identified healthy according to the American College of Obstetricians and Gynecologists (ACOG) (Appendix A), 2) GA between 20–24 weeks, 3) Latina, 4) proficient in English, and 5) have access to the internet and smart phone.

### Sample size

This is a pilot study where there is no prior information to base the sample size on. In this situation, the recommendation from the literature suggests a sample size of 12 as being appropriate for the purposes of preliminary data collection and analysis (Julious, 2005). According to recent statistics, approximately 10% of the low-risk pregnancies still have a chance of progressing to complications (Rajbanshi et al., 2020). Considering additional 15% as the possible attrition rate, the final sample size was considered to be *n* = 16.

### Measures

#### HRV

The Oura Ring was used to continuously capture HRV data in participants during the study. The Oura Ring is a water-proof smart ring made of highly scratch-resistant materials, including zirconia (Zr02), with a medical-grade, nickel-free, 100% non-allergenic, non-metallic inner molding. It is wireless (Bluetooth) and weighs approximately 3–4 g, depending on the size of the ring. A full battery charge would be enough to capture signals for 5–7 consecutive days (Kinnunen et al., 2020). The sensors embedded in the inner layer of the ring include a PPG, 3D accelerometer, gyroscope, and negative thermal coefficient (NTC) body temperature sensor. The ring has been used before in human subjects (de Zambotti et al., 2019; Maijala et al., 2019; Altini and Kinnunen, 2021). The Oura Ring also comes in different sizes, which can be selected based on personal comfort. See “data procedure section” for more details. The HRV metrics such as NN interval, RMSSD, SDANN, SDNN, pNN50, LF, HF, and LF/HF were supposed to be extracted from the interbeat interval (IBI) captured by sensors. The relevant machine learning coding was supposed to be applied in order to obtain the HRV metrics from IBI data.

#### Healthiness criteria assessment

To assure the healthy status of the subjects based on ACOG criteria, EPIC and REDCap were applied to constantly monitor for possible risk factors and confounding factors that have the potential to threaten the healthy situation of the participants.

EPIC is a platform that is considered to be the information backbone for a majority of the largest integrated health systems in the world. EPIC provided information regarding the obstetric, medical, and sociodemographic details of the participants that were required for eligibility assessment as well as weekly monitoring for the possible complications. Given that prenatal care visits become more frequent, with weekly visits toward the end of pregnancy, it indeed aligns perfectly with our choice of weekly monitoring for identifying potential complications updated in the EPIC system. This frequency ensures that we are in sync with the evolving needs of the participants and the timing of their prenatal care, making it a practical and well-aligned approach.

We also used REDCap to subjectively assess 1) the potential mental-related risk factors, identified by the ACOG, such as mental distress (stress, anxiety, and depression), and 2) lifestyle-related factors (activity, acute stress, sleep, and diet) as possible predictive factors for HRV.

For mental distress, we used the Patient Health Questionnaire-2 (PHQ-2), Perceived Stress Scale 4 (PSS-4), and Generalized Anxiety Disorder 2 (GAD-2) to assess depression, stress, and anxiety, respectively. These measures for stress, anxiety, and depression have all been validated on pregnant individuals with an education level of at least junior high school in urban and rural settings (Essiben et al., 2018; Thomas et al., 2019; Whittle et al., 2019).

The short version of the Personal Health Questionnaire depression scale (PHQ-2) is a 4-point scale and includes two items to measure the “frequency of depressed mood” over the last 2 weeks. This scale includes four points ranging from 0 (not at all) to 3 (nearly every day). The possible highest and lowest total scores are 0 and 6, respectively. The cutoff point of the PHQ-2 is 3, and a score greater than 3 is considered to be major depression. The PHQ-2 has a good reliability (alpha Cronbach of 0.92), sensitivity (74%), and specificity (60%) in the United States (Gelaye et al., 2016) and was used for the US pregnant population (Bennett et al., 2008; Slavin et al., 2020).

The GAD-2 is a 4-point scale containing two items to assess “anxiety” and “worry” over the last 2 weeks. The possible responses vary ranging from 0 (not at all) to 3 (nearly every day). The total score ranges from 0 to 6; the higher the score, the severe the anxiety. The cutoff point of the GAD-2 is 3, and a score equal to and greater than 3 is considered to be major anxiety. The GAD-2 has 86% sensitivity and 83% specificity for the diagnosis of generalized anxiety disorder applying in the US population (Kroenke et al., 2007).

The PSS-4 is a 5-point scale and includes four items of “control,” “confidence,” “satisfaction,” and “overcoming the difficulties” in the last month. The possible responses varied ranging from 0 (almost never) to 4 (very often). The possible highest and lowest total scores are 0 and 20, respectively, with the higher score showing higher stress. The cutoff point of the PSS-4 is 6, and a score equal to and greater than 6 is considered to be major stress. This 4-item scale has internal reliability with an alpha Cronbach of 0.69 among the US population (Warttig et al., 2013).

The frequency of mental distress assessment depends on the ability of the determined measurement scale to capture the potential mental distress. For example, the PSS-4 has been designed to assess stress over the last month; thus, monthly assessment is considered for stress assessment. For the PHQ-2 and GAD-2, biweekly assessment was considered. The measurement frequency is specified in Table 1.

**TABLE 1:** Measurement frequency for the measures.

	Assessment tool	Pregnancy (week)																	*Postartum* (week)
		22–24	25	26	27	28	29	30	31	32	33	34	35	36	37	38	39	40	1
ANS (HRV) assessment	HRV	Continuous measurement via Oura/weekly capturing assessment																	
ACOG-identified risk factors	PSS-4	-+	--	--	--	-+	--	--	--	-+	--	--	--	-+	--	--	--	-+	**= +**
	GAD-2	-+	--	-+	--	-+	--	-+	--	-+	--	-+	--	-+	--	-+	--	-+	**= +**
	PHQ-2	-+	--	-+	--	-+	--	-+	--	-+	--	-+	--	-+	--	-+	--	-+	**= +**
	EPIC-reported risk factors	-+	-+	-+	-+	-+	-+	-+	-+	-+	-+	-+	-+	-+	-+	+	-+	++	**= +**
Descriptive characteristics	Sociodemographic characteristics	++	--	--	--	--	--	--	--	--	--	--	--	--	--	--	--	--	--
Potential predictors	Lifestyle-related factors in Oura	Continuous measurement via Oura/weekly capturing assessment																	
Potential predictors	Lifestyle-related factor assessment using daily surveys	Daily assessment																	

For lifestyle-related elements, we applied a daily survey questionnaire to assess factors such as activity, sleep, stress, and diet reported by participants. The questions were distributed at the end of the day before they go to bed. The daily assessments offered several advantages. It allowed us to capture participants’ perceptions of their lifestyle on a daily basis when the information was still fresh in their memory. This approach minimized the risk of forgetting details due to extended time intervals between assessments, which can often lead to less-accurate responses.

The lifestyle-related factors were also assessed objectively using the Oura Ring. These data included sleep metrics (e.g., total sleep time, sleep efficiency, time spent in different sleep stages (light, deep, and REM), sleep latency (time taken to fall asleep and the number of awakenings during the night), activity metrics (e.g., steps taken, active calories burned, and total active time), body temperature, heart rate (e.g., resting heart rate, average heart rate, and heart rate variability [HRV]), respiratory rate, O2 saturation, and readiness and recovery scores. The Oura-derived objective data were monitored weekly to identify and address the possible technical issues in capturing data. If abnormal data (meaningless pattern) or shortage of data recorded (less than 30%) is observed, we contacted the participants for the possible technical issues or barriers that might be the potential reasons. The decision to adopt a weekly monitoring schedule was intentional for two primary reasons. First and foremost, it corresponds with the maximum syncing time interval recommended by Oura to maintain data within the app, ensuring that un-synced data are not treated as missing data. Second, the choice of a weekly assessment frequency strikes a balance, allowing for timely monitoring without imposing substantial burdens on both researchers and participants.

Descriptive information performed once at the beginning of the study to describe the population.

### Study procedure

**FIGURE 1:**
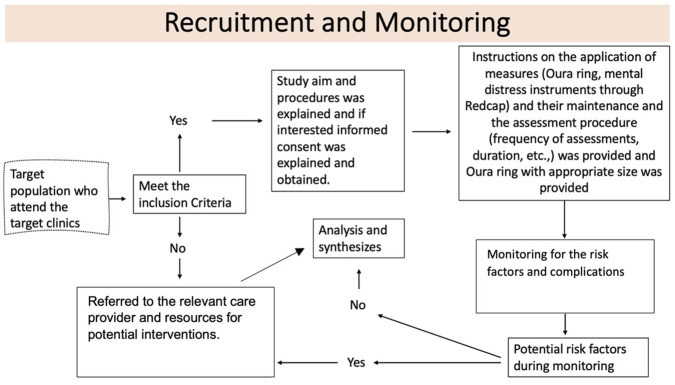
Recruitment procedure.

Following the IRB approval, a purposive sampling was conducted to screen the potential candidates in the selected clinic. The eligibility criteria were checked through the EPIC system. After establishing the eligibility criteria, prospective participants were identified and subsequently contacted in the clinic to confirm their eligibility and assess their interest in the study participation. If they met the inclusion criteria and consented to participate, they proceeded to the monitoring phase of the study. If no complications or risk factors were reported during monitoring, the participants were deemed healthy and proceeded to the analysis phase (Figure 1). However, if complications occurred, the participants were referred to the appropriate healthcare provider for potential interventions and still considered for comparison analysis as the exploratory aim.

### Data cleaning

The data cleaning process involves three steps. First, data screening is performed to identify four types of abnormalities: missing data, inconsistencies and outliers, unusual patterns of distributions, and unexpected analysis results. The second step is the diagnostic phase, where each abnormal value is investigated to determine if it is erroneous, a true extreme, or has an unknown cause. For example, unrealistic age and BMI values were checked in the EPIC system to obtain the correct values. Additionally, extreme values for education were found to be due to participants having multiple degrees. Last, the treatment phase involves resolving the identified abnormal data by correcting, deleting, or leaving them unchanged. In this study, impossible values were replaced with the true values obtained from documents, and missing data were imputed using prediction techniques. However, for participant #13, who had unexplainable negative and out-of-range sleep and HRV data, the enormous values could not be treated, leading to their exclusion from the analysis.

### Data analysis

We applied supervised techniques (e.g., the hierarchical linear regression/mixed effect model (HLM) and random forest regression (RFR)) to address the study aim in R and Python software. The HLM enabled us to understand the association between HRV and time across the participants. Even though it is not generalizable and statistically reliable due to lower sample size, we also came up with the exploratory result comparing a complicated case and healthy participants in a weekly basis using the *t*-test. Using other supervised techniques such as RFR, we tried to understand the potential predictors and predicting features’ importance for HRV magnitude during pregnancy.

## Results

### Sampling

**FIGURE 2:**
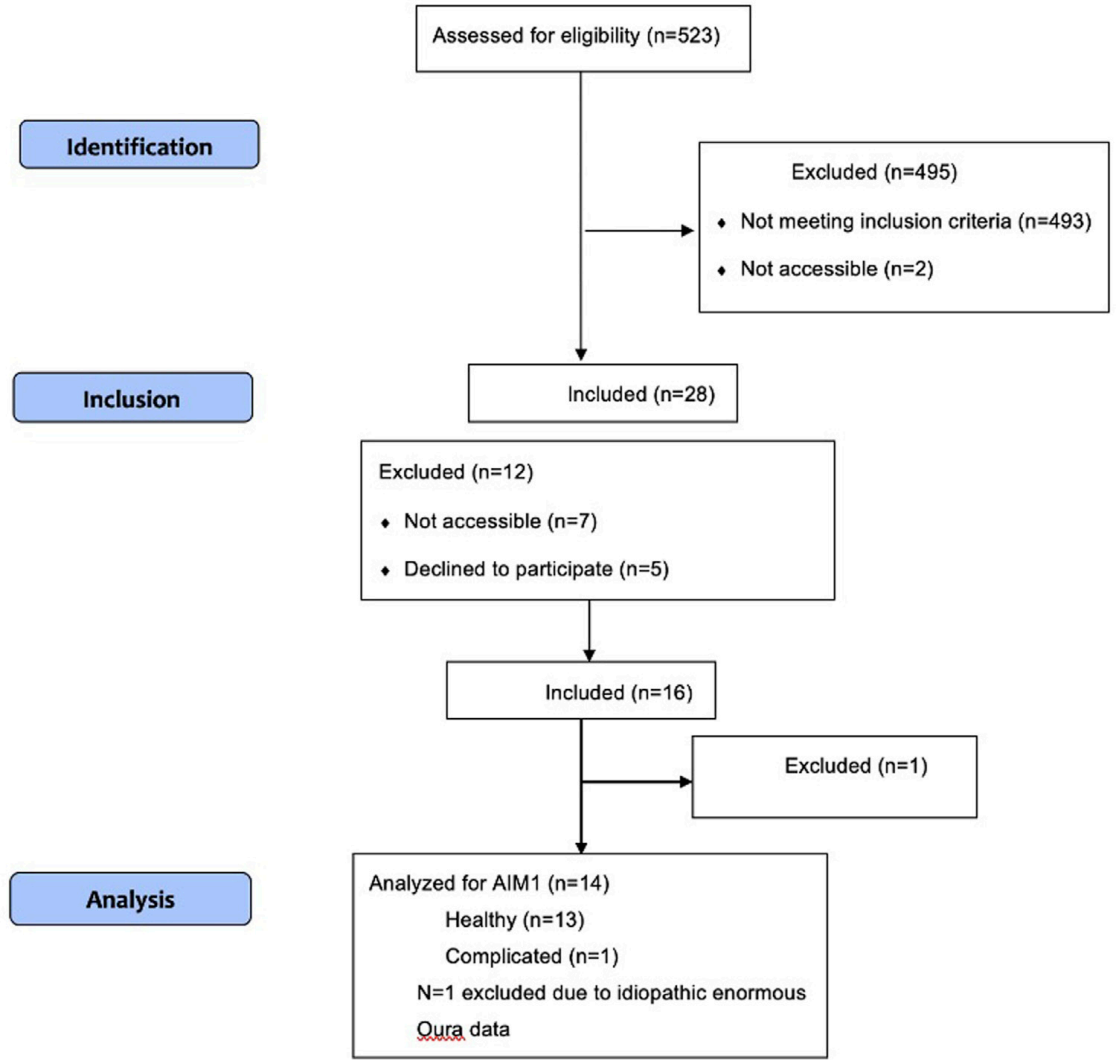
STROBE chart.

The recruitment started on August 21st and continued until November 16th in the Manchester Clinic affiliated to the UCI to enroll all required 16 participants into the study based on the inclusion criteria. We assessed 523 individuals for potential eligibility in the EPIC system. *N* = 493 did not meet the inclusion criteria, and *N* = 2 individuals were receiving prenatal service outside the facility where the study recruitment was taking place, thus excluded. *N* = 28 individuals were identified as eligible for the study. Out of this, *N* = 7 participants did not make their prenatal care appointment as they were scheduled. *N* = 5 declined to participate in the study (*n* = 3 needed their husband’s permission; *n* = 2 brought no reason). Finally, *n* = 16 participants enrolled into the study (please see Figure 2 for the STROBE chart). One of the participants declined to continue in the middle of the study since she had broken her hand and was prohibited to wear any jewelry per her care provider’s prescription. In addition, *N* = 1 participant experienced a complicated pregnancy (placental abruption) at 34 weeks. As a result, *N* = 14 healthy participants completed the study. However, instead of completely excluding the complicated pregnancy case, we included it in the analysis for exploratory purposes. This allowed us to compare and gain insights into how it differed from the healthy participants in terms of HRV magnitude. In the data cleaning phase, *N* = 1 participant was excluded due to the idiopathic enormous Oura data. Thus, *N* = 13 accounting for healthy participants and *N* = 1 accounting for a complicated participant were considered for the final analysis.

#### Descriptive information

Sociodemographic characteristics of the study population (*n* = 14), upon enrollment, were assessed using a demographic questionnaire. As represented in Table 2, age, height, weight, and BMI are in the healthy category based on the ACOG criteria for healthy pregnancy. All the participants completed at least junior high school in terms of education. Approximately 50% of the participants (7/14) had income under $35 k, 64% (9/14) were in their second pregnancy, and all had given birth at least once before.

**TABLE 2:** Sociodemographic distribution.

Variable		Finding
Age (years) (mean [SD])		31.4 [3.85]
Height (cm) (mean [SD])		167 [6.53]
Weight (kg) (mean [SD])		68 [6.83]
BMI (mean [SD])		24.22 [2.06]
Education (years) (mean [SD])		17.73 [5.39]
Income level (number [%])	$18 - 34.999K	7 [50%]
	$35 – 99.999 K	5 [36%]
	$100 – 199.999 K	2 [14%]
Gravidity (number [%])	2	9 [64%]
	3	2 [14%]
	4	3 [22%]
Parity (number [%])	1	9 [64%]
	2	2 [14%]
	3	3 [22%]

### HRV analysis

Unfortunately, we were not able to obtain IBI data due to the policy change of the Oura company in providing such data. Several efforts were made to request for reconsideration, but no improvement was achieved. Thus, we used RMSSD, the existing HRV metric provided by the Oura company.

#### Hierarchical linear regression/mixed-effects model

We used the HLM to examine the relationship between time as the independent variable and RMSSD as the dependent variable to determine whether the time could impact and predict HRV magnitude.

**FIGURE 3:**
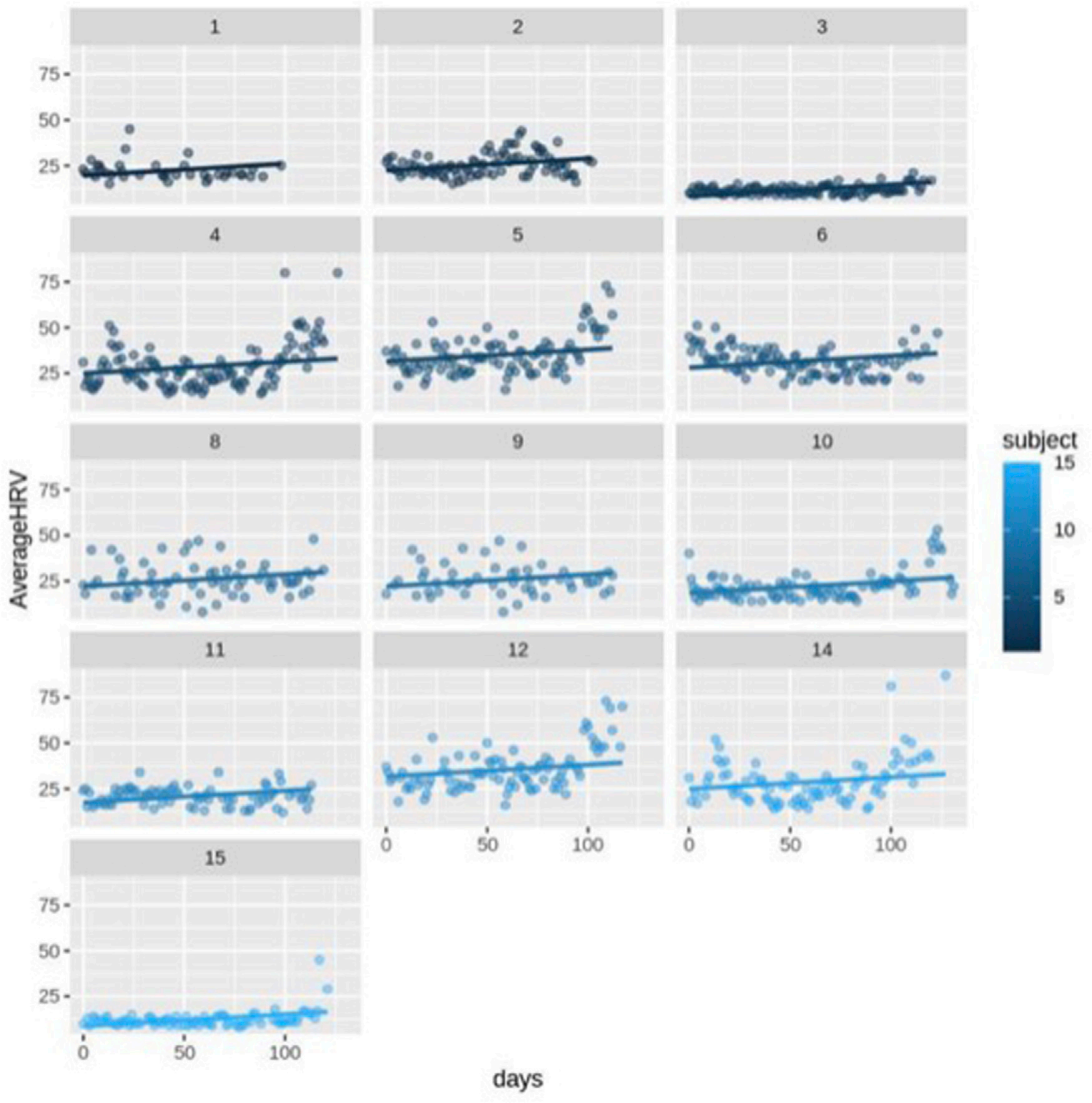
Scatter plot for HRV changes with different intercepts and the same slope over time (day) for healthy subjects (*n* = 13).

**FIGURE 4:**
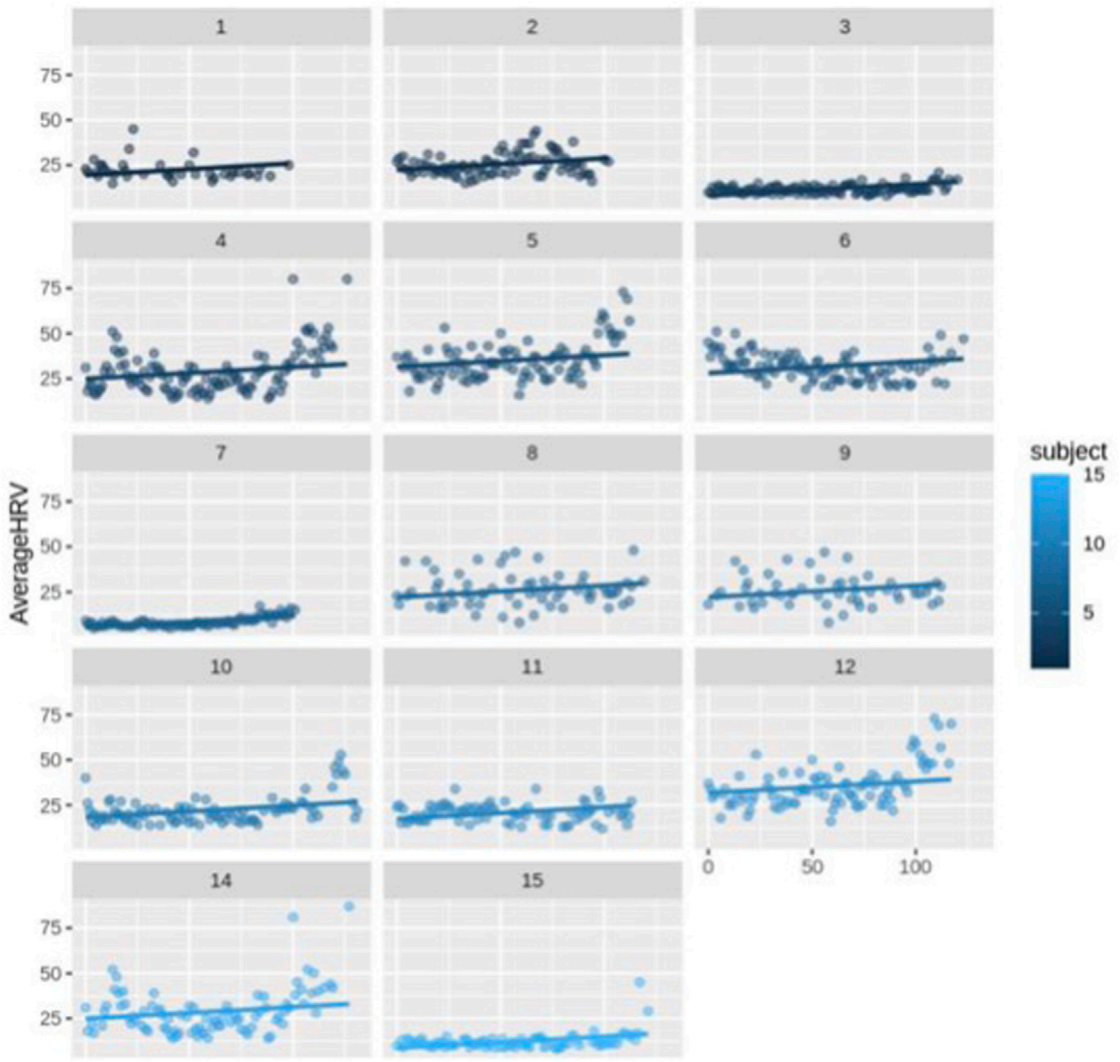
Scatter plot for HRV changes with different intercepts and the same slope over time (day) for each subject (*n* = 14).

First, we ran the model for all the participants (*N* = 14) including healthy (*N* = 13) and complicated cases (*N* = 1). The fixed-effects estimates indicated that the intercept (average HRV) at day 0 is 2.037e-01 (20.37) and the slope (change in average HRV per day) is 0.06427, and both estimates are statistically significant (*p* < 0.0001). It means for each increase in day, HRV increases by 0.06427 across the participants (Figure 3; Figure 4).

The random error estimate indicated that there is a substantial variation in the intercepts across subjects with the standard deviation of 8.315. The residual standard deviation was 7.976. The correlation of fixed effects showed that there is a negative correlation between the intercept and the slope, which means that subjects with higher intercepts tend to have smaller slopes. Overall, this model suggested that there is a significant positive relationship between days and average HRV, and that there is substantial variation in average intercepts across all subjects.

Then, we excluded the *N* = 1 complicated case and ran the model for only healthy subjects. This model still indicated a significant positive connection between days and average HRV, as well as notable variability in average intercepts among the healthy subjects. The fixed-effects estimates indicated that the intercept at day 0 is 21.54 and the slope is 0.06442, and both estimates are statistically significant, *p*-value = 1.28e-07 and *p*-value < 2e-16, respectively (*p* < 0.0001). As it can be seen, excluding the complicated subject did not affect the significance of the HRV changes over time. It means for each increase in day, HRV still increases by approximately 0.06 in the healthy participants. The estimation of the random error reveals considerable variability in the intercepts among subjects, with a standard deviation of 7.35. The residual standard deviation is 8.28. The correlation of fixed effects demonstrates a negative association between the intercept and the slope, indicating that subjects with higher intercepts tend to have smaller slopes.

#### Random forest regression

In this study, we also used RFR to build regression models to predict HRV using multi-channel time series data. RFR is a type of ensemble learning method that combines multiple decision trees to make predictors. RFR works by randomly selecting subsets of the features and building decision trees on each subset and then combining the results to make final predictors. All subjective (survey) and objective (Oura) data were applied in the RFR model to understand the importance of them in predicting the HRV magnitude in pregnancy.

**FIGURE 5:**
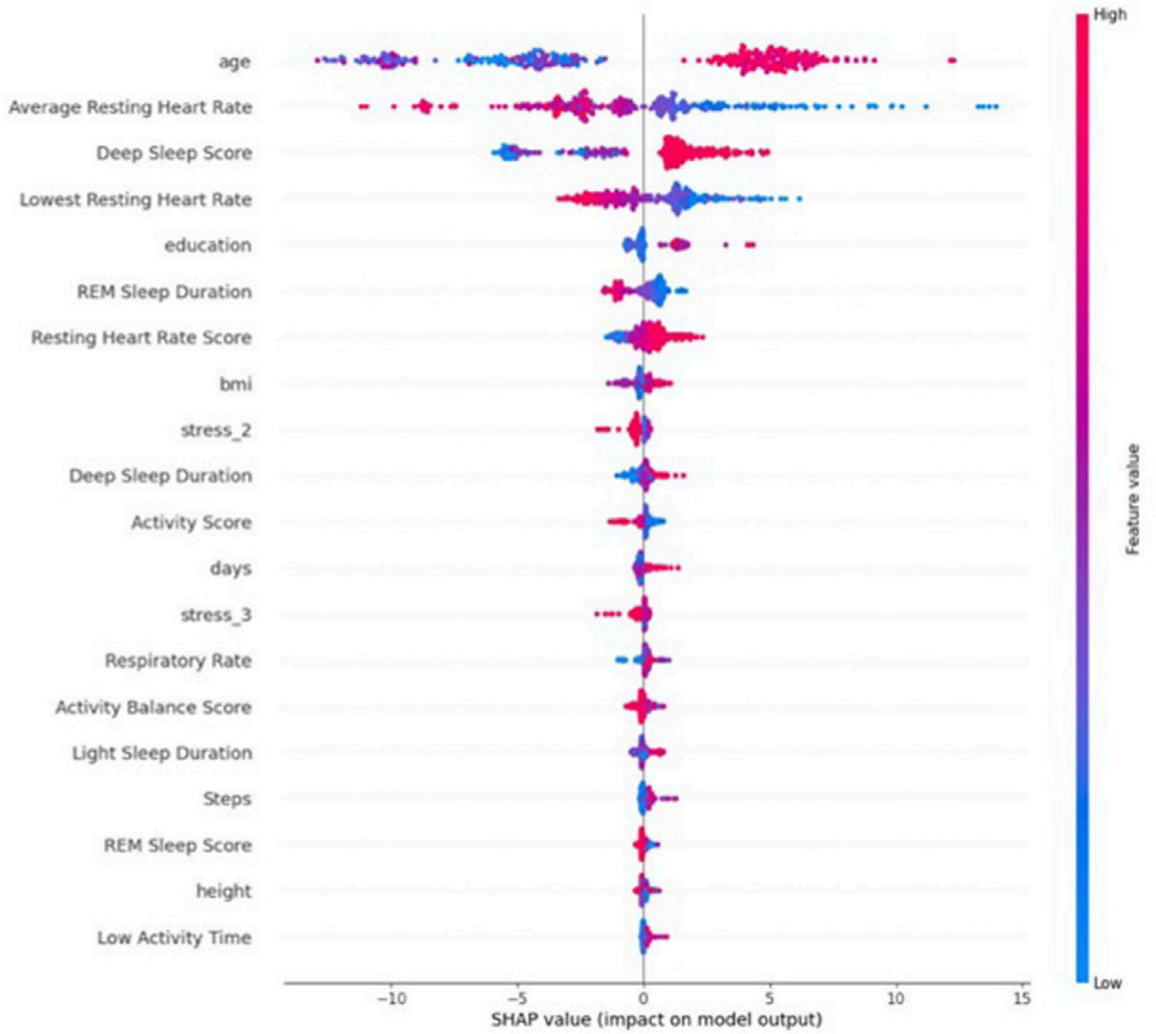
Shapley value for average HRV.

As shown in Figure 5, the most important predictive features are age, average resting heart rate, deep sleep score, lowest resting heart rate, education, REM sleep duration, resting heart rate score, BMI, mental stress, deep sleep duration, activity score, time, respiratory rate, activity balance score, light sleep duration, steps, REM sleep score, and low-activity time. Some of these features have a positive correlation with HRV. For example, the higher the age, deep sleep score, education, resting heart rate score, and time (gestational age), the higher the HRV magnitude. Other features either had a negative (average resting heart rate, lowest resting heart rate, and REM sleep duration) or non-clear/multi-directional (step, activity score, BMI, respiratory rate, height, and steps) correlation with HRV. However, we should always be mindful that these correlations that come from the machine learning techniques such as RFR are inherently non-linear. This means that the relationship between the input variables (features) and the output variable (RMSSD, in this case) can be complex and not easily captured by simple linear equations. As a result, the associations between individual features and the outcome predicted by a machine learning model may not always be explicitly positive or negative. In other words, the impact of a particular feature on the predicted outcome may depend on its interactions with other features or on other factors that are not easily observable. This is where techniques like Shapley values can be useful. Shapley values are a way to measure the contribution of individual features to the predicted outcome of a machine learning model, taking into account their interactions with other features. They provide a more nuanced understanding of the relationships between features and outcomes, which can be helpful in identifying key factors that influence the predicted outcome. By generating Shapley value figures for the features in the RFR model, we can gain insights into the relative importance of different features and how they contribute to the overall prediction of HRV value. This can help us identify potential areas for further investigation or intervention and can also provide a more comprehensive understanding of the factors that influence HRV.

**FIGURE 6:**
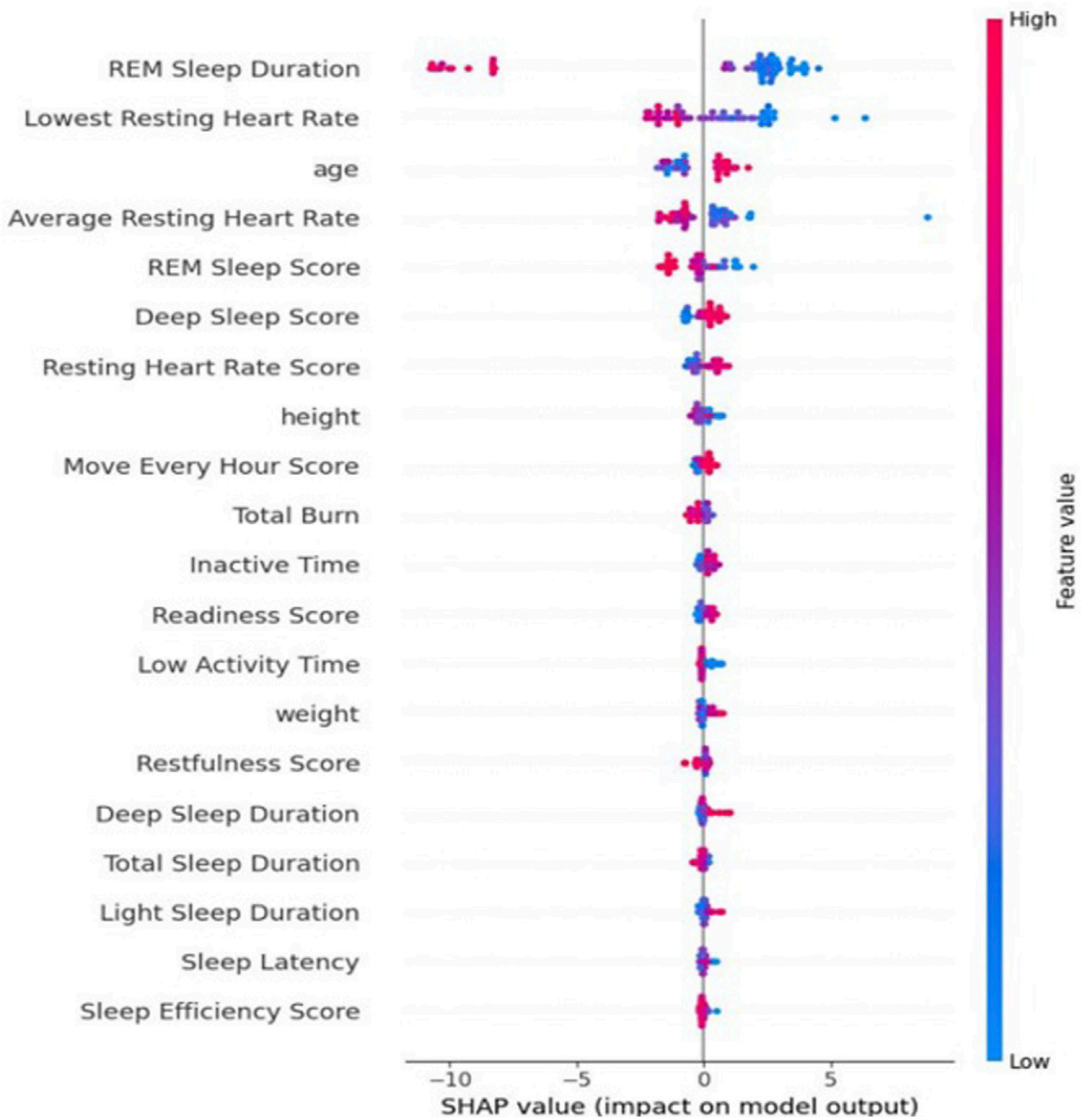
Shapley value for the average HRV intercept.

**FIGURE 7:**
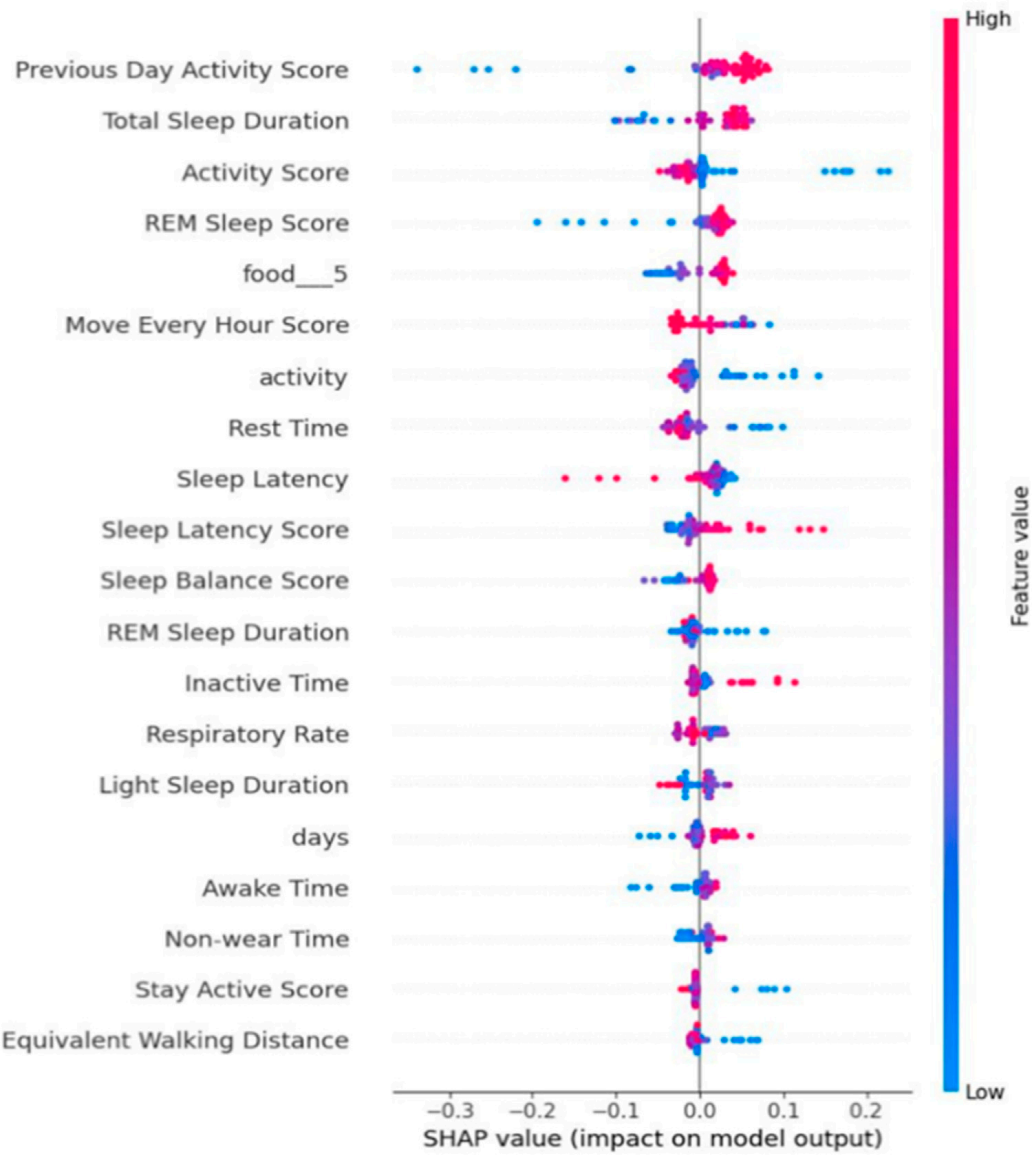
Shapley value for the average HRV slope.

Figure 6 and Figure 7 indicate the important predictive features for the average HRV intercept and slope, respectively. As can be seen, features such as age, height, sleep, activity, and heart rate still are among important predictive features for average HRV intercept. Weight, restfulness, and total burn are features that seem to predict the average HRV intercept but not the average HRV value and average HRV slope. The average HRV slope shares common predictive features with average HRV and HRV intercept in the majority of the features. Food is the feature that appeared in the average HRV slope but not in the average HRV intercept and average HRV value.

The accuracy of the model was assessed using the root mean square error (RMSE) metric which is the difference between observed and predicted values in regression analysis. RMSE for the RFR model in the current study was 4.70 which is lower than the global baseline of RMSE (12.28), indicating that this model is more accurate than the global average prediction accuracy for healthy population.

### Exploratory analysis

Although statistically unreliable due to small sample size, we compared the HRV magnitude between subjects who kept the healthy status by the end of the study and the complicated case. This may give an insight on the possible difference between healthy and complicated pregnant individuals.

### *t*-test for weekly comparison of the participants

**FIGURE 8:**
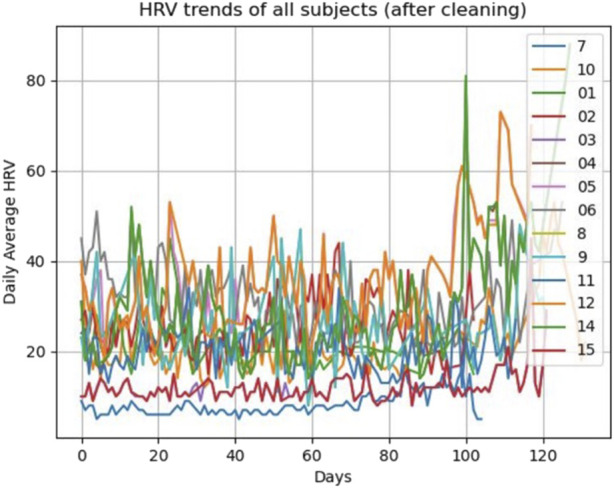
HRV pattern for all 14 subjects during the study.

**FIGURE 9:**
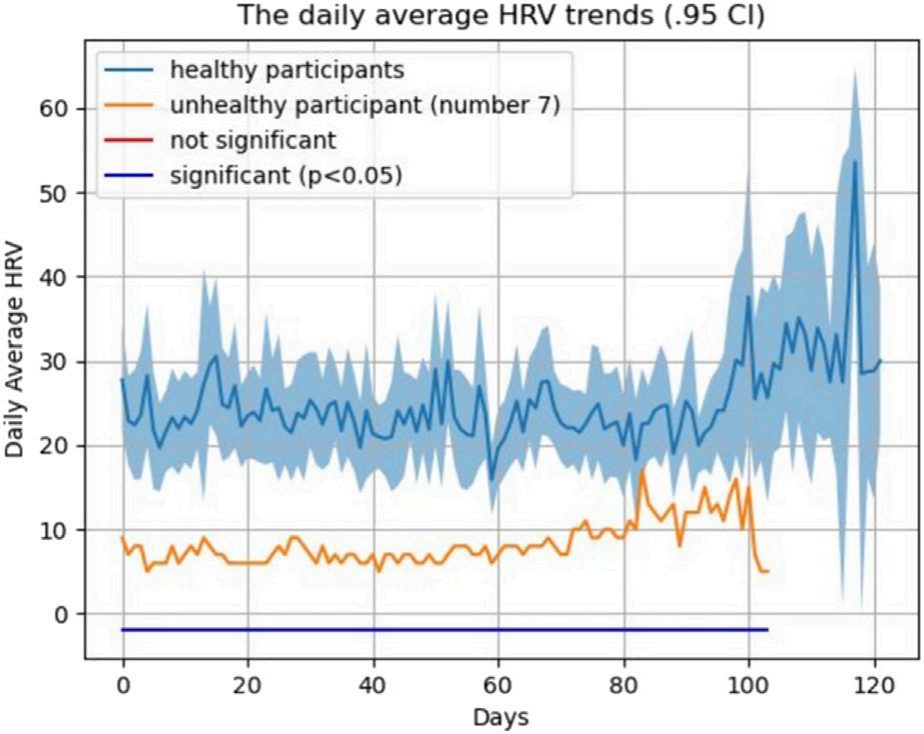
HRV pattern for healthy and unhealthy subjects during the study (daily).

**FIGURE 10:**
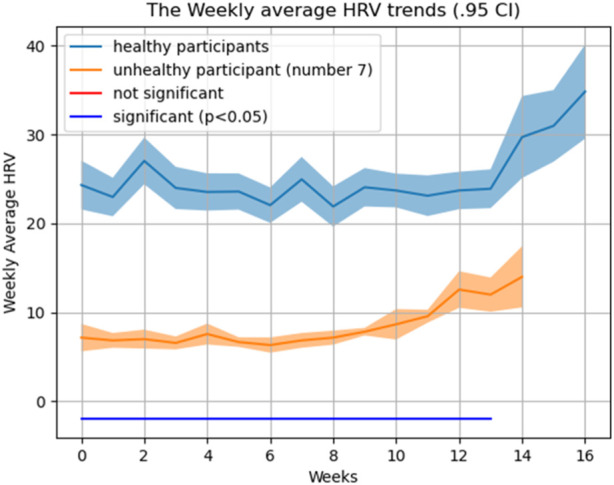
HRV pattern for healthy and unhealthy subjects during the study (weekly).

A *t*-test was used to compare the weekly HRV between the healthy (*N* = 13) and non-healthy (*N* = 1) participants. We calculated weekly average HRV across healthy participant to be able to compare it with complicated subject (subject #7). As shown in Figure 8, Figure 9, and Figure 10, an average HRV in healthy and non-healthy subjects is statistically significant (*p* < 0.05) during 17 weeks of the study length when assessed daily and weekly.

**FIGURE 11:**
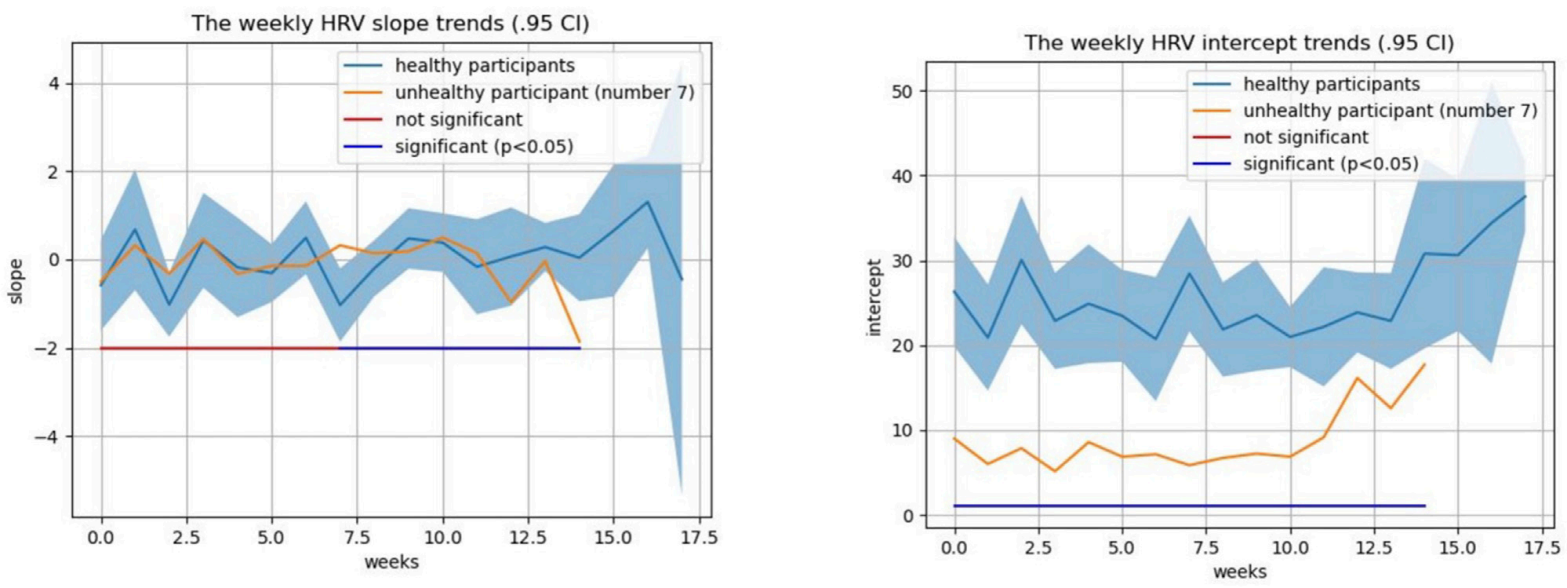
Average HRV intercept and slope comparison between healthy and complicated subjects.

In addition to the weekly average HRV value, we considered the weekly average HRV slope and intercept to see how their changes occur in addition to the weekly average HRV. After week 7 of the study (31st gestational week), the weekly HRV slope difference between healthy and non-healthy subjects becomes significant (*p* < 0.05) (Figure 11). The average weekly intercept is statistically different (*p* < 0.05) between the healthy and non-healthy participants during the entire study (Figure 11).

## Discussion

As discussed earlier, the ANS plays a crucial role in adapting to the unique demands of pregnancy and preventing complications. However, current methods for assessing the ANS function have limitations. To ensure a healthy pregnancy, it is important to accurately assess the ANS function. The aim of this study was to understand the pattern of different HRV metrics extracted from IBI data for healthy pregnant participants. However, we were not able to address the aim thoroughly due to the policy change of the Oura company in providing IBI data. Thus, we conducted planned analyses on the existing HRV (RMSSD) data provided by the Oura. We applied supervised machine learning techniques such as the HLM and RFR, as well as the *t*-test, to address the research question. The results of HLM analysis showed a significant positive relationship between time and average HRV regardless of being healthy or complicated, indicating that HRV increases over time significantly. The model also revealed substantial individual variation in intercepts and slopes between all subjects. The findings of comparison analysis using the *t*-test method indicated that the average HRV of healthy and unhealthy subjects differed significantly during the 17 weeks of the study. Overall, the results suggested that although the HRV is significantly different between the healthy and unhealthy participants, time could be a predictor of HRV magnitude in both groups. Additionally, the findings from the RFR analysis suggested that HRV could be influenced by factors beyond time alone, such as lifestyle, mental health, and sociodemographic factors. This indicates that HRV is not solely determined by time, but it also has the potential to be influenced and regulated. Therefore, if the existing literature’s association between HRV and pregnancy complications is validated, it may be possible to predict and address these complications by making relevant lifestyle adjustments and accommodations. Among the effective factors, sleep, activity, BMI, diet, and mental stress were the most significant predictive features for HRV.

To the best of our knowledge, there is only one study that assessed HRV changes longitudinally during pregnancy. Sarhaddi et al. (2022) conducted a study to assess trends in the heart rate and HRV parameters during pregnancy and the 3-month *postpartum* period. Their findings indicated that RMSSD is higher in the third trimester than that in the second trimester, which aligns with our results. However, these results conflict with the findings from the studies that assessed HRV non-continuously during pregnancy using short-term and episodic assessments (Sharifiheris et al., 2022).

In the systematic review conducted by Sharifiheris et al. (2022), HRV was operationalized in three main groups of the frequency domain, time domain, and non-linear domain. In the reviewed studies, RMSSD, which is characterized as time domain metrics, tends to decrease during pregnancy, as opposed to the findings of the current study. However, the studies with such a finding often suffered from serious limitations. For example, the assessment duration was 5–10 min in the majority of the studies. This short-term assessment duration threatens the reliability of the findings; as according to the literature, any duration less than 24 h is unable to represent ANS function.

We could not find any study that concerned with predictive factors of HRV, other than time, in pregnant women. However, the literature supports the finding of this study that HRV is not solely under impact of time. Due to its complex task, the ANS has various relationships with internal and external elements. For example, age (Jandackova et al., 2016; Geovanini et al., 2020), prenatal alcohol consumption (Karpyak et al., 2014; Jurczyk et al., 2019), smoking (Harte et al., 2013; Bodin et al., 2017; Murgia et al., 2019), drug use (Nayak et al., 2020; Lu et al., 2022; Qiu et al., 2023), and BMI (Rodrigues and Quarto, 2018; Triggiani et al., 2019) have been shown to have a correlation with HRV. Although demographic factors such as age cannot be controlled, other factors that are mainly lifestyle-related factors are adjustable by the individual. The current study’s results align with these findings indicating that lifestyle-related factors such as sleep, activity, diet, and mental stress affect HRV changes over time.

Understanding HRV seems to be crucial since, 1) as discussed earlier, it has potential to be predicted and, thus, can be adjusted for better outcomes, 2) more importantly, it can predict common pregnancy complications. For instance, there are a few studies that showed HRV can predict maternal mental stress and emotional states during pregnancy with high accuracy (Cao et al., 2022; Li et al., 2022). In addition to these studies that were concerned with the emotional and mental outcome of HRV, there are studies that applied HRV classification for physical pregnancy-induced complications such as hypertensive disorders. For example, Tejera et al. (2011) conducted a study to understand the accuracy of HRV indexes in classifying normal, hypertensive, and preeclamptic pregnancies using the machine learning approach. The results showed that the applied artificial neural network (ANN) model was able to accurately classify the three pregnancy groups including normotensive, hypertensive, and preeclamptic groups based on HRV measures.

The collective evidence presented in these studies demonstrates the potential of HRV within the context of pregnancy and predicting various pregnancy outcomes. These findings suggest that HRV data can provide valuable information about the physiological and psychological states of pregnant women and may have important implications for the diagnosis and management of various pregnancy-related conditions.

Furthermore, the studies’ results highlight the importance of incorporating HRV data into routine prenatal care to provide a more comprehensive understanding of maternal health. HRV analysis has been shown to offer valuable insight into fetal growth, preeclampsia, gestational diabetes, and other pregnancy-related conditions. Various studies have shown that HRV analysis can provide early warning signs of complications, allowing for timely interventions to improve maternal and fetal health outcomes. In our study, low RMSSD in placental abruption as compared to healthy individuals showed HRV’s potential for prediction application. Placental abruption is a significant contributing factor to preterm birth, a condition associated with sympathetic predominance of the ANS in the existing literature (Aye et al., 2018; Jasinski et al., 2022). Placental abruption involves the partial or complete separation of the placenta from the uterine wall before the onset of labor. Understanding the temporal relationship between placental abruption and ANS dysfunction is a complex endeavor, as ANS dysregulation can play multiple roles. It can act as a preceding factor explaining placental abruption or serve as a compensatory response following placental abruption to maintain homeostasis.

In the first scenario, placental abruption may arise as a consequence of hypertensive disorders, which have been consistently linked to an increased risk of placental abruption. This association is primarily attributed to the impact of hypertensive disorders on placental function through vasoconstriction. This vasoconstriction, the narrowing of blood vessels, ultimately reduces blood flow to the uterus and placenta, potentially leading to their separation from the uterine wall.

In the second scenario, placental abruption can result in maternal hypovolemia, characterized by a reduced blood volume due to bleeding following the abruption. This condition triggers physiological responses that impact the ANS, leading to an overactivation of the sympathetic branch. Indeed, in an effort to compensate for hypovolemia, the sympathetic nervous system may become dominant, potentially resulting in a reduction in parasympathetic activity.

Given its application in the literature, HRV should be given careful consideration in prenatal care. Additional research is necessary to confirm these findings and identify the most optimal approaches for integrating HRV analysis into standard prenatal care practices.

## Limitations and strengths

This study prioritized a minoritized group, pregnant Latina women, who are at a greater risk of developing pregnancy complications, and thus, better understanding of potential mechanisms of action of complications is critical. The application of smart technology also enabled us to communicate with our participants easily and improve engagement. We also considered strict inclusion and exclusion criteria which increases the internal validity of the study. Since more than 80% of pregnancies are identified as low risk, this also increases the generalizability of our study and, thus, external validity.

Although we tried to minimize the limitations, our study still suffered from a few limitations. We were not able to address our first aim which was extracting and tracking the various HRV patterns using IBI data due to the policy change of Oura in providing such data. This taught us, for the future study(s), to identify the target company thoroughly and obtain a signed contract promising the data access. Lack of access to various metrics of HRV disabled us to represent the ANS comprehensively. We had to rely on the single HRV metric, RMSSD, which is not enough to reflect sympathetic–parasympathetic activity. In addition, this is a pilot study for which the main aim is an investigation or small-scale trial to assess the feasibility and potential of a larger study. Thus, the statistical findings may not be reliable in this small trial, specifically the analysis that compares the healthy and complicated pregnancies as we only had one complicated case that statistically is not able to represent the complicated pregnancies. However, these analyses still give insights regarding the potential changes of HRV in healthy pregnant women during the second and third trimesters of pregnancy and its possible predictors.

## Conclusion

In this study, we were able to capture HRV changes over time and investigate the patterns and trends that occur during pregnancy. This allowed us to demonstrate that HRV increases over time during pregnancy regardless of the health status of the pregnancy. We were also able to identify specific gestational weeks at which the HRV magnitude was significantly different between healthy and unhealthy subjects. Although statistically unreliable, we showed that there is a difference between complicated and healthy pregnancy in terms of the average HRV value.

RFR analysis also allowed us to identify potential predictors of HRV during pregnancy in addition to time. These predictors included, but not limited to, age, BMI, weight, height, activity, sleep, food, and mental stress.

## Data Availability

The raw data supporting the conclusion of this article will be made available by the authors, without undue reservation.

## Appendix A

**TABLE A1:** Criteria for healthy pregnancy in the American College of Obstetricians and Gynecologists.

I. Social/personal	II. Obstetric history	III. Medical and family history	IV. Present pregnancy
Maternal age (<17, >35 years)	Gravida (1 or >5)	Diabetes	Pre-pregnancy BMI ≤25
Education (<6 years)	Para (0 or >5)	Hypertension	Height <145 cm
Marital status (unmarried)	Abortions (>2)	Renal diseases	Pregnancy-induced hypertension
Economic status (poor)	Miscarriages (1+)	Cardiovascular diseases (e.g., phlebitis)	Bleeding
Smoking, alcohol consumer, or drug user	Fetal deaths (≥1)		Pre-eclampsia
Domestic violence	Bleeding in T3	Blood diseases	Multiple pregnancy
	Stillbirths (≥1)	Endocrine diseases	Abnormal fetal presentation
	Previous cesarean (≥1)	No medication use except for perinatal vitamins and folate	Gestational diabetes mellitus
	Preterm deliveries (≥1)	STDs	Placenta previa
	Birth weights (<2500 gr)	Respiratory diseases	Cervix insufficiency
	Infant deaths (≥1)	Central nervous diseases	An Rh-negative woman with an Rh-positive husband who is not willing to take RhoGAM
	Toxemia (≥1)	Abdominal visceral disorders	
	Birth defects (≥1)	Genetic or congenital disorder	
		Autoimmune disease	
		Infectious diseases	
		Mental illness (mental distress such as stress, anxiety, and depression)*	

*PHQ-2, PSS-4, and GAD-2 were used to assess depression, stress, and anxiety, respectively.

## References

[B1] Altini M. Kinnunen H. (2021). The promise of sleep: a multi-sensor approach for accurate sleep stage detection using the Oura ring. Sensors 21 (13), 4302. 10.3390/s21134302 34201861 PMC8271886

[B2] Aye C. Y. Lewandowski A. J. Oster J. Upton R. Davis E. Kenworthy Y. (2018). Neonatal autonomic function after pregnancy complications and early cardiovascular development. Pediatr. Res. 84 (1), 85–91. 10.1038/s41390-018-0021-0 29795212 PMC6086328

[B3] Bennett I. M. Coco A. Coyne J. C. Mitchell A. J. Nicholson J. Johnson E. (2008). Efficiency of a two-item pre-screen to reduce the burden of depression screening in pregnancy and postpartum: an IMPLICIT network study. J. Am. Board Fam. Med. 21 (4), 317–325. 10.3122/jabfm.2008.04.080048 18612058 PMC3606919

[B4] Bodin F. McIntyre K. M. Schwartz J. E. McKinley P. S. Cardetti C. Shapiro P. A. (2017). The association of cigarette smoking with high frequency heart rate variability: an ecological momentary assessment study. Psychosom. Med. 79 (9), 1045–1050. 10.1097/PSY.0000000000000507 28731984 PMC5675783

[B5] Braeken M. A. R. I. J. K. E. (2014). Psychological functioning and the autonomic nervous system during pregnancy. Doctoral dissertation, PhD thesis. Tilburg, the Netherlands: Tilburg University. Impact on mother and child.

[B6] Bruno R. M. Ghiadoni L. Seravalle G. Dell'Oro R. Taddei S. Grassi G. (2012). Sympathetic regulation of vascular function in health and disease. Front. physiology 3, 284. 10.3389/fphys.2012.00284 PMC3429057 22934037

[B7] Cao R. Rahmani A. M. Lindsay K. L. (2022). Prenatal stress assessment using heart rate variability and salivary cortisol: a machine learning-based approach. Plos one 17 (9), e0274298. 10.1371/journal.pone.0274298 36084123 PMC9462678

[B8] de Zambotti M. Rosas L. Colrain I. M. Baker F. C. (2019). The sleep of the ring: comparison of the OURA sleep tracker against polysomnography. Behav. Sleep. Med. 17 (2), 124–136. 10.1080/15402002.2017.1300587 28323455 PMC6095823

[B9] Duong H. T. H. Tadesse G. A. Nhat P. T. H. Van Hao N. Prince J. Duong T. D. (2020). Heart rate variability as an indicator of autonomic nervous system disturbance in tetanus. Am. J. Trop. Med. Hyg. 102 (2), 403–407. 10.4269/ajtmh.19-0720 31833471 PMC7008337

[B10] Ernst G. (2017). Heart-rate variability—more than heart beats? Front. public health 5, 240. 10.3389/fpubh.2017.00240 28955705 PMC5600971

[B11] Essiben F. Um E. M. N. Ojong S. Gimnwi F. Olen K. Nana P. N. (2018). GAD-7 and PHQ-9 measurement of perinatal anxiety and depression in women with hypertensive disorders of pregnancy in Yaounde, Cameroon. Int. J. Reproduction, Contracept. Obstetrics Gynecol. 7 (6), 2069. 10.18203/2320-1770.ijrcog20182312

[B12] Gelaye B. Wilson I. Berhane H. Y. Deyessa N. Bahretibeb Y. Wondimagegn D. (2016). Diagnostic validity of the patient health questionnaire-2 (PHQ-2) among Ethiopian adults. Compr. psychiatry 70, 216–221. 10.1016/j.comppsych.2016.07.011 27567282 PMC5108453

[B13] Geovanini G. R. Vasques E. R. de Oliveira Alvim R. Mill J. G. Andreão R. V. Vasques B. K. (2020). Age and sex differences in heart rate variability and vagal specific patterns–Baependi heart study. Glob. Heart 15 (1), 71. 10.5334/gh.873 33150136 PMC7583712

[B14] Harte C. B. Liverant G. I. Sloan D. M. Kamholz B. W. Rosebrock L. E. Fava M. (2013). Association between smoking and heart rate variability among individuals with depression. Ann. Behav. Med. 46 (1), 73–80. 10.1007/s12160-013-9476-8 23436273

[B15] Howell E. A. Egorova N. N. Janevic T. Balbierz M. A. Zeitlin J. Hebert P. L. (2017). Severe maternal morbidity among Hispanic women in New York City: investigation of health disparities. Obstetrics Gynecol. 129 (2), 285–294. 10.1097/AOG.0000000000001864 PMC5380443 28079772

[B16] Jandackova V. K. Scholes S. Britton A. Steptoe A. (2016). Are changes in heart rate variability in middle‐aged and older people normative or caused by pathological conditions? Findings from a large population‐based longitudinal cohort study. J. Am. Heart Assoc. 5 (2), e002365. 10.1161/JAHA.115.002365 26873682 PMC4802439

[B17] Jasinski S. R. Rowan S. Presby D. M. Claydon E. Capodilupo E. R. (2022). Wearable-derived maternal heart rate variability as A novel digital biomarker of preterm birth. medRxiv, 2022–2111. 10.1371/journal.pone.0295899 PMC10829979 38295026

[B18] Johnson J. O. (2019). “Autonomic nervous system: physiology,” in Pharmacology and physiology for anesthesia (Elsevier), 270–281.

[B19] Julious S. A. (2005). Sample size of 12 per group rule of thumb for a pilot study. Pharm. Statistics J. Appl. Statistics Pharm. Industry 4 (4), 287–291. 10.1002/pst.185

[B20] Jurczyk M. Dyląg K. A. Skowron K. Gil K. (2019). Prenatal alcohol exposure and autonomic nervous system dysfunction: a review article. Folia Medica Cracoviensia 59 (3), 15–21. 10.24425/fmc.2019.131132 31891356

[B21] Karpyak V. M. Romanowicz M. Schmidt J. E. Lewis K. A. Bostwick J. M. (2014). Characteristics of heart rate variability in alcohol‐dependent subjects and nondependent chronic alcohol users. Alcohol. Clin. Exp. Res. 38 (1), 9–26. 10.1111/acer.12270 24117482

[B22] Kataoka K. Tomiya Y. Sakamoto A. Kamada Y. Hiramatsu Y. Nakatsuka M. (2015). Altered autonomic nervous system activity in women with unexplained recurrent pregnancy loss. J. Obstetrics Gynaecol. Res. 41 (6), 912–918. 10.1111/jog.12653 25546149

[B23] Kinnunen H. Rantanen A. Kenttä T. Koskimäki H. (2020). Feasible assessment of recovery and cardiovascular health: accuracy of nocturnal HR and HRV assessed via ring PPG in comparison to medical grade ECG. Physiol. Meas. 41 (4), 04NT01. 10.1088/1361-6579/ab840a 32217820

[B24] Kroenke K. Spitzer R. L. Williams J. B. Monahan P. O. Löwe B. (2007). Anxiety disorders in primary care: prevalence, impairment, comorbidity, and detection. Ann. Intern. Med. 146 (5), 317–325. 10.7326/0003-4819-146-5-200703060-00004 17339617

[B25] Li X. Ono C. Warita N. Shoji T. Nakagawa T. Usukura H. (2022). Heart rate information-based machine learning prediction of emotions among pregnant women. Front. Psychiatry 12, 799029. 10.3389/fpsyt.2021.799029 35153864 PMC8830335

[B26] Lu M. K. Chou L. W. Chang K. M. Lee H. Y. Kang H. J. (2022). Substance misuse decreases heart rate variability. J. Sensors 2022, 1–7. 10.1155/2022/3222839

[B27] Maijala A. Kinnunen H. Koskimäki H. Jämsä T. Kangas M. (2019). Nocturnal finger skin temperature in menstrual cycle tracking: ambulatory pilot study using a wearable Oura ring. BMC Women's Health 19 (1), 150–210. 10.1186/s12905-019-0844-9 31783840 PMC6883568

[B28] Murgia F. Melotti R. Foco L. Gögele M. Meraviglia V. Motta B. (2019). Effects of smoking status, history and intensity on heart rate variability in the general population: the CHRIS study. PLoS One 14 (4), e0215053. 10.1371/journal.pone.0215053 30964923 PMC6456196

[B29] Nayak S. K. Pradhan B. K. Banerjee I. Pal K. (2020). Analysis of heart rate variability to understand the effect of cannabis consumption on Indian male paddy-field workers. Biomed. Signal Process. Control 62, 102072. 10.1016/j.bspc.2020.102072

[B30] Peng R. C. Zhou X. L. Lin W. H. Zhang Y. T. (2015). Extraction of heart rate variability from smartphone photoplethysmograms. Comput. Math. methods Med. 2015, 516826. 10.1155/2015/516826 25685174 PMC4309304

[B31] Petersen E. E. Davis N. L. Goodman D. Cox S. Syverson C. Seed K. (2019). Racial/ethnic disparities in pregnancy-related deaths—United States, 2007–2016. Morb. Mortal. Wkly. Rep. 68 (35), 762–765. 10.15585/mmwr.mm6835a3 PMC6730892 31487273

[B32] Qiu H. Zhang H. Han D. D. Derakhshandeh R. Wang X. Goyal N. (2023). Increased vulnerability to atrial and ventricular arrhythmias caused by different types of inhaled tobacco or marijuana products. Heart rhythm. 20 (1), 76–86. 10.1016/j.hrthm.2022.09.021 36603937 PMC10006068

[B33] Rajbanshi S. Norhayati M. N. Nik Hazlina N. H. (2020). High-risk pregnancies and their association with severe maternal morbidity in Nepal: a prospective cohort study. PloS one 15 (12), e0244072. 10.1371/journal.pone.0244072 33370361 PMC7769286

[B34] Rodrigues T. S. Quarto L. J. G. (2018). Body mass index may influence heart rate variability. Arq. Bras. Cardiol. 111, 640–642. 10.5935/abc.20180201 30365689 PMC6199506

[B35] Sarhaddi F. Azimi I. Axelin A. Niela-Vilen H. Liljeberg P. Rahmani A. M. (2022). Trends in heart rate and heart rate variability during pregnancy and the 3-month postpartum period: continuous monitoring in a free-living context. JMIR mHealth uHealth 10 (6), e33458. 10.2196/33458 35657667 PMC9206203

[B36] Sharifiheris Z. Rahmani A. Onwuka J. Bender M. (2022). The utilization of heart rate variability for autonomic nervous system assessment in healthy pregnant women: systematic review. JMIR Bioinforma. Biotechnol. 3 (1), e36791. 10.2196/36791 PMC11135217 38935943

[B37] Slavin V. Creedy D. K. Gamble J. (2020). Comparison of screening accuracy of the Patient Health Questionnaire-2 using two case-identification methods during pregnancy and postpartum. BMC Pregnancy Childbirth 20, 211–215. 10.1186/s12884-020-02891-2 32290813 PMC7158032

[B38] Soma-Pillay P. Nelson-Piercy C. Tolppanen H. Mebazaa A. (2016). Physiological changes in pregnancy: review articles. Cardiovasc. J. Afr. 27 (2), 89–94. 10.5830/CVJA-2016-021 27213856 PMC4928162

[B39] Tejera E. Jose areias M. Rodrigues A. Ramoa A. Manuel nieto-villar J. Rebelo I. (2011). Artificial neural network for normal, hypertensive, and preeclamptic pregnancy classification using maternal heart rate variability indexes. J. Maternal-Fetal Neonatal Med. 24 (9), 1147–1151. 10.3109/14767058.2010.545916 21250912

[B40] Thomas J. L. Lewis J. B. Martinez I. Cunningham S. D. Siddique M. Tobin J. N. (2019). Associations between intimate partner violence profiles and mental health among low-income, urban pregnant adolescents. BMC pregnancy childbirth 19 (1), 120. 10.1186/s12884-019-2256-0 31023259 PMC6485079

[B41] Triggiani A. I. Valenzano A. Trimigno V. Di Palma A. Moscatelli F. Cibelli G. (2019). Heart rate variability reduction is related to a high amount of visceral adiposity in healthy young women. PloS one 14 (9), e0223058. 10.1371/journal.pone.0223058 31553779 PMC6760781

[B42] Warttig S. L. Forshaw M. J. South J. White A. K. (2013). New, normative, English-sample data for the short form perceived stress scale (PSS-4). J. health Psychol. 18 (12), 1617–1628. 10.1177/1359105313508346 24155195

[B43] Waxenbaum J. A. Reddy V. Varacallo M. (2019). Anatomy, autonomic nervous system. 30969667

[B44] Whittle H. J. Sheira L. A. Wolfe W. R. Frongillo E. A. Palar K. Merenstein D. (2019). Food insecurity is associated with anxiety, stress, and symptoms of posttraumatic stress disorder in a cohort of women with or at risk of HIV in the United States. J. Nutr. 149 (8), 1393–1403. 10.1093/jn/nxz093 31127819 PMC6675617

[B45] Yousif D. Bellos I. Penzlin A. I. Hijazi M. M. Illigens B. M. W. Pinter A. (2019). Autonomic dysfunction in preeclampsia: a systematic review. Front. Neurology 10, 816. 10.3389/fneur.2019.00816 PMC6691156 31447757

[B46] Ziemssen T. Siepmann T. (2019). The investigation of the cardiovascular and sudomotor autonomic nervous system—a review. Front. neurology 10, 53. 10.3389/fneur.2019.00053 PMC6380109 30809183

[B47] Zygmunt A. Stanczyk J. (2010). Methods of evaluation of autonomic nervous system function. Archives Med. Sci. 6 (1), 11–18. 10.5114/aoms.2010.13500 PMC3278937 22371714

